# Reacquisition of cocaine conditioned place preference and its inhibition by previous social interaction preferentially affect D1-medium spiny neurons in the accumbens corridor

**DOI:** 10.3389/fnbeh.2014.00317

**Published:** 2014-09-24

**Authors:** Janine M. Prast, Aurelia Schardl, Christoph Schwarzer, Georg Dechant, Alois Saria, Gerald Zernig

**Affiliations:** ^1^Experimental Psychiatry Unit, Innsbruck Medical UniversityInnsbruck, Austria; ^2^Department of Pharmacology, Innsbruck Medical UniversityInnsbruck, Austria; ^3^Institute for Neuroscience, Innsbruck Medical UniversityInnsbruck, Austria; ^4^Department of Psychology, Leopold-Franzens University of InnsbruckInnsbruck, Austria

**Keywords:** cocaine, social interaction, conditioned place preference, accumbens, septum, island of Calleja, diagonal band, D1 medium spiny neurons

## Abstract

We investigated if counterconditioning with dyadic (i.e., one-to-one) social interaction, a strong inhibitor of the subsequent reacquisition of cocaine conditioned place preference (CPP), differentially modulates the activity of the diverse brain regions oriented along a mediolateral corridor reaching from the interhemispheric sulcus to the anterior commissure, i.e., the nucleus of the vertical limb of the diagonal band, the medial septal nucleus, the major island of Calleja, the intermediate part of the lateral septal nucleus, and the medial accumbens shell and core. We also investigated the involvement of the lateral accumbens core and the dorsal caudate putamen. The anterior cingulate 1 (Cg1) region served as a negative control. Contrary to our expectations, we found that all regions of the accumbens corridor showed increased expression of the early growth response protein 1 (EGR1, Zif268) in rats 2 h after reacquisition of CPP for cocaine after a history of cocaine CPP acquisition and extinction. Previous counterconditioning with dyadic social interaction inhibited both the reacquisition of cocaine CPP and the activation of the whole accumbens corridor. EGR1 activation was predominantly found in dynorphin-labeled cells, i.e., presumably D1 receptor-expressing medium spiny neurons (D1-MSNs), with D2-MSNs (immunolabeled with an anti-DRD2 antibody) being less affected. Cholinergic interneurons or GABAergic interneurons positive for parvalbumin, neuropeptide Y or calretinin were not involved in these CPP-related EGR1 changes. Glial cells did not show any EGR1 expression either. The present findings could be of relevance for the therapy of impaired social interaction in substance use disorders, depression, psychosis, and autism spectrum disorders.

## Introduction

The nucleus accumbens has long been a major target for studies on the rewarding effects of drugs of abuse or physiological reinforcers, whereas the brain regions medial of the medial accumbens shell have received less attention, although they mediate a number of aspects of reward: As early as 1963, it was found that electrical self-stimulation of the septal area produces intensely pleasurable effects in humans and serves as an effective reinforcer (Heath, 1963). The lateral septum is activated by both the conditioned and direct pharmacologic effects of cocaine (Brown et al., 1992) and extensively interconnects with the medial accumbens shell (Zahm et al., 2013). As a last example, amphetamine increases c-Fos expression and histone H3 phosphorylation in the major island of Calleja (Rotllant and Armario, 2012). All these findings suggest that previous studies using probes aimed at the medial accumbens region and supposedly only reporting “specific accumbal” effects may in effect have tapped these regions as well, bearing in mind that, e.g., a microdialysis probe has an *inner* diameter of as much as 240 μm (Zernig and Fibiger, 1998) and may easily sample from a corridor as wide as 500 μm. The present study therefore set out to investigate the effects of cocaine—both its noncontingent (i.e., direct pharmacologic) and contingent (i.e., conditioned or “psychologic”) effects—and the effects of a previous history of counterconditioning with dyadic (i.e., one-to-one) social interaction on cocaine's conditioned effects in all brain regions medial of the anterior commissure, while paying close attention to the exact spatial distribution of the signal.

These regions can be distinguished by a number of differences in histoarchitecture, neuropil staining (Paxinos et al., 2009) and connectivity (Heimer et al., 1991, 1997; Zahm, 2008). In particular, the medial accumbens core (AcbCm) which shares a lot of similarities with the dorsal striatum (Heimer et al., 1997; Ikemoto, 2007; Haber and Knutson, 2010) and the medial accumbens shell (AcbShm), which is considered part of the “extended amygdala” (Heimer et al., 1997), differentially mediate many aspects of reward (Pontieri et al., 1995; Berlanga et al., 2003; Acquas et al., 2007; Zavala et al., 2008). Accordingly, we had shown that conditioned place preference (CPP) for cocaine is mediated by the core, whereas CPP for dyadic social interaction between sex- and weight matched early adult male Sprague–Dawley rats is mediated by the shell (Fritz et al., 2011a). However, our previous study suffered from the same weakness that jeopardizes a number of core-vs.-shell investigations in that we had defined “core” and “shell” only with respect to their proximity to the anterior commissure as the imaginary center of this sphere-like accumbal region (Paxinos and Watson, 2007; Paxinos et al., 2009), while neglecting the fact that all striatal pathways, including the Acb, are arranged along a (dorso)lateral to (ventro)medial gradient (Ikemoto, 2007; Haber and Knutson, 2010). Accordingly, in our previous study (Fritz et al., 2011a) we had lesioned a shell portion medial of the anterior commissure (AcbShm) and a core region around the anterior commissure, affecting both a medial portion of the core (AcbCm) that lies immediately adjacent to the AcbShm and a lateral core subregion (AcbCl) that is contiguous with the caudate putamen (CPu) proper. To correct for that inconsistency, we defined a mediolateral corridor stretching from the interhemispheric sulcus to the anterior commissure and systematically divided it into 250 μm bins that well correspond to the following regions (Paxinos et al., 2009): The nucleus of the vertical limb of the diagonal band and the medial septal nucleus (VDB+MS), the major island of Calleja and the intermediate part of the lateral septal nucleus (ICjM+LSI), the AcbShm, AcbCm, and AcbCl (Brog et al., 1993; Zahm, 2008; Zahm et al., 2013) and investigated their activation in an animal model of cocaine relapse prevention by previous social interaction that we had developed (Fritz et al., 2011b; Zernig et al., 2013). As the above regions differ considerably with respect to their histoarchitecture and connectivity, we expected to see pronounced differences in the various regions' response to such strikingly different rewards as cocaine vs. social interaction, an expectation that was soundly refuted by the findings detailed below. Therapeutically, our findings could be of relevance as many drug addicts suffer from impaired social interaction and would greatly profit from a reorientation of their preference away from the drug of abuse toward social interaction (Zernig et al., 2007, 2013). Of note, impaired social interaction is not only found in substance use disorders but is also a major challenge in the treatment of depression, psychosis, or autism spectrum disorders (American Psychiatric Association, 1994).

## Materials and methods

### Subjects

Early adult male Sprague–Dawley rats (150–250 g, corresponding to an age of 6–8 weeks) were obtained from the Research Institute of Laboratory Animal Breeding of the Medical University Vienna (Himberg, Austria) and were single housed at 24°C for 1 week before the start of the experiment. The animals received *ad libitum* access to tap water and pellet chow, and a 12-h light/dark cycle with lights on from 0800 to 2000 h was maintained. All animals were treated according to high ethical and scientific standards of the European Union. The present experiments were approved by the Austrian National Animal Experiment Ethics Committee.

### Place conditioning procedure

#### Housing conditions and CPP apparatus

Conditioning was conducted in a homemade three compartment apparatus (CPP box) as described before (Kummer et al., 2011). All behavioral tests were videorecorded and analyzed offline with hand timers for the time spent in each compartment. Experiments were conducted during the light period of the cycle. Masking background noise was generated by a continuously running high efficiency particulate air (HEPA) antiallergen filter box. The CPP box was placed directly beneath a neon tube (58 W, 1 m distance).

#### Naïve animals, noncontingent cocaine treatment, and cocaine CPP training

**Figure 1:**
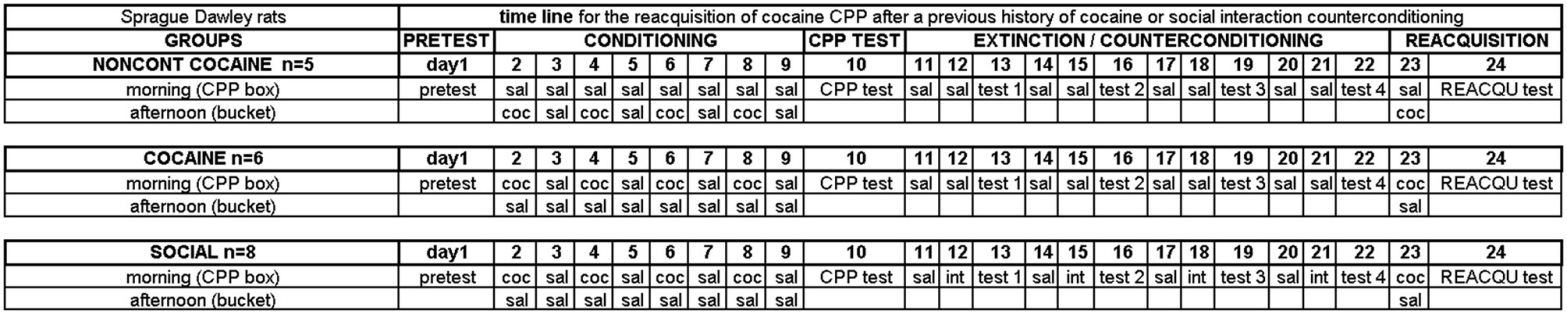
**Experimental timeline**. Experimental groups and group sizes are listed on the left. See Materials and Methods Section for more details.

After 1 week of single housing, i.e., at an age of 7–9 weeks, one group of animals (naïve, *n* = 8) was sacrificed before undergoing any further treatment to investigate EGR1 expression in naïve animals. Thus, the naïve group was 24 days younger than all other groups on the day of perfusion. This age difference may have affected basal intracellular signaling activity, although we are not aware of any systemic study addressing this issue. The three other treatment groups comprised rats that were injected noncontingently with cocaine (NONCONT, *n* = 5), trained for cocaine CPP and extinguished with saline (COCAINE, *n* = 6), or trained for cocaine CPP followed by social interaction counterconditioning (SOCIAL, *n* = 8). The detailed timeline of the behavioral training is shown in Figure 1. First, all animals were tested for their pretest bias on day 1 which was declared if the rat spent one second or more in one of the two conditioning compartments during pretest (total test or training session length was always 900 s). Cocaine injections were paired with the initially nonpreferred side. On the following day, cocaine CPP acquisition training was started by injecting COCAINE animals intraperitoneally (i.p.) with cocaine HCl corresponding to 15 mg/kg pure base (a gift to Gerald Zernig from the National Institute on Drug Abuse, NIDA) or saline (1 ml/kg) in an alternate day design in the morning outside of the CPP box and by putting each animal into the respective compartment inside the CPP box (day 2–9). In the afternoon, i.e., at least 6 h after the CPP training in the morning, all cocaine CPP animals received a saline injection in a context clearly different from the CPP box, i.e., they were injected i.p. with saline and placed into a bedding-filled bucket for 15 min before being put back into the home cage. The NONCONT group received i.p. saline injections before being put in either compartment of the CPP box during CPP training (i.e., were trained for saline vs. saline) and, in the afternoon, noncontingently (i.e., not in close temporal association with any CPP conditioning procedure) received the same number of saline or cocaine injections in the same alternate day design (day 2–9) as the COCAINE group. This procedure assured that the NONCONT animals could not associate any compartment of the CPP box with the interoceptive effects of cocaine, thus controlling for (a) the pharmacologic effect of cocaine and (b) handling and i.p. injection effects. Cocaine CPP preference was tested on day 10 in a 15-min test session in a cocaine-free state.

#### Extinction of cocaine CPP or social interaction counterconditioning

After acquisition of cocaine CPP, animals in the COCAINE group (*n* = 6) were extinguished with saline, whereas animals in the SOCIAL group (*n* = 8) were counterconditioned with dyadic (i.e., one-to-one) social interaction (day 11–22) in that they received the opportunity to engage in dyadic social interaction in the non-cocaine-associated compartment (Fritz et al., 2011b). Effective counterconditioning was declared if the preference of the animal shifted from the cocaine associated compartment to the social interaction associated compartment (see Figure 3 of Fritz et al., 2011b). One day after the CPP test, the COCAINE animals and the NONCONT group received a saline injection in the cocaine compartment followed by a saline injection in the saline compartment the following day and a CPP test 1 day later (test 1–test 4). The animals of the SOCIAL group were also injected with saline in the compartment previously paired with cocaine on day 1 of the 3-day cycle and received an i.p. saline injection before being put into previously saline-paired compartment on day 2 but were subjected to a 15-min social interaction with a sex- and weight-matched conspecific in this compartment. On day 3, they were tested for CPP in a cocaine- and interaction-free state. This 3-day cycle of training-training-test was repeated three more times for all groups (day 11–22). The 3-day cycle had originally been used by us to investigate the time course of the extinction and social interaction counterconditioning (Fritz et al., 2011b). In the present study, we stuck to this schedule to render the present findings comparable to our previous ones.

#### Reacquisition of cocaine CPP

On day 23, an additional cocaine conditioning trial took place during which COCAINE or SOCIAL animals were injected with cocaine before being put into the previously cocaine-paired compartment of the CPP box in the morning. These animals received a saline injection in the afternoon before being placed in a bucket (to keep the total number of injections the same as for the NONCONT group). In contrast, NONCONT animals were saline-injected in the initially (i.e., pretest-determined) non-preferred compartment in the morning, followed by a cocaine injection and a bucket placement in the afternoon. 24 h later, i.e., in a cocaine-free state, all animals were tested for the reacquisition of cocaine CPP (day 24, REACQU test). Two hours after the start of the 15-min cocaine CPP reacquisition test, i.e., at the expected peak of EGR1 protein expression (Zangenehpour and Chaudhuri, 2002), animals were deeply anesthetized with isoflurane and their brains were processed for immunohistochemistry.

### Definition of counting areas

To precisely define the borders of the regions in the accumbens corridor at different anteroposterior (AP) positions with respect to bregma we used the chemoarchitectonic atlas of Paxinos et al. (2009) in which the core and shell subregion of the accumbens are visualized by their differential calbindin staining. Abbreviations follow these authors' convention (Paxinos and Watson, 2007) except for the “m” (for “medial”) and “l” (for “lateral”) extensions that we added to their terms “AcbSh” or “AcbC” to designate the location of these Acb subregions relative to the anterior commissure. Because we used slices only from the AP positions +2.2 to +1.0 mm relative to bregma (Meredith et al., 1989), we measured the width (i.e., mediolateral extension) of the AcbCm and the AcbShm at the height of the anterior commissure at three different AP positions, i.e., at +1.928, +1.616, and +1.304 mm, obtaining a mean width of 250 μm for each of these Acb subregions. Accordingly, we divided the whole accumbens corridor into 250 μm bins and were able to distinguish the following regions (from medial to lateral): VDB+MS, ICjM+LSI, AcbShm, and AcbCm (Prast et al., 2012). **Figure 6A** shows a schematic graph of the accumbens corridor and its defined borders. A 250 μm strip immediately lateral of the anterior commissure represented the AcbCl. The dorsal CPu was also analyzed, the dorsal edge of the counting rectangle forming a segment with the dorsalmost curvature of the corpus callosum. The anterior cingulate region Cg1 (Paxinos and Watson, 2007) served as a negative control, as we had previously shown (Fritz et al., 2011b) that a history of social interaction counterconditioning does not affect the cocaine CPP reacquisition-associated activation in this region.

### Immunohistochemistry

Animals were intracardially perfused with 0.1 mol/l phosphate buffered saline (PBS) followed by 4% (w/v) paraformaldehyde (PFA) dissolved in PBS (pH 7.4). Brains were removed and postfixed in 4% PFA overnight, cryoprotected in PBS containing 10% sucrose (w/v) for 1 day and in 30% sucrose PBS at 8°C until the brains sank, shock-frozen in isopentane (at a temperature between −35°C and −39°C) and stored at −80°C until sectioning. All serial brain sections (25 μm) were cut using a Cryostat (Leica). Sections were stored in PBS containing 0.1% sodium azide at 8°C until immunolabeling. A total of three slices per rat for each marker were randomly chosen and slices were free-floated for 48 h at 8°C in 50 mM Tris-buffered saline (TBS; pH 7.4) containing 0.1% Triton-X-100 (TBS-T) and 2% BSA with the primary antibody against EGR1 (1:1000, Santa Cruz Biotechnology, sc-189) and a second primary antibody against dynorphin (goat polyclonal anti-DYN; 1:50, Santa Cruz Biotechnology, sc-46313) to label D1-MSNs (but see the Discussion Section), or against the dopamine D2 receptor (mouse polyclonal anti-DRD2, 1:50, Santa Cruz Biotechnology, sc-5303) to label D2-MSNs. The marker used for cholinergic interneurons was choline acetyltransferase (goat polyclonal anti-ChAT, 1:166, Millipore, AB144p). The different GABAergic interneuron types were identified by their immunoreactivity for parvalbumin (goat polyclonal anti-PV, 1:1000, Swant, PVG-214), calretinin (goat polyclonal anti-CR, 1:1000, Swant, CG1) or neuropeptide Y (goat polyclonal anti-NPY, 1:500, Novus, NBP1-46535). Sections were washed three times for 5 min each in TBS-T and were incubated at 90–100°C in a 10 mM citrate buffer, pH 6.0, for 7 min for antigen retrieval. After a TBS-T wash, slices were incubated for 30 min in TBS containing 50 mM glycine, followed by another wash in TBS-T (3 × 5 min) and a 1 h incubation in TBS-T containing 2% BSA and 10% normal donkey serum (Millipore, S30) or 7% normal donkey and 3% normal goat serum (Vector Labs, S-1000) depending on the primary antibodies used. For the investigation of the involvement of neuronal vs. glial cells in the observed behavioral changes, we performed double immunohistochemistry for EGR1 and a mouse polyclonal antibody against the neuron-specific nuclear protein NeuN (Mullen et al., 1992, anti-NeuN, 1:200, Millipore, MAB377) or one of the following glial markers: Mouse polyclonal anti glial fibrillary acidic protein (anti-GFAP; 1:200, Santa Cruz Biotechnology, sc-33673) to identify astrocytes (Wang et al., 2013), mouse polyclonal myelin basic protein (anti-MBP; 1:200, Santa Cruz Biotechnology, sc-71546) for oligodendrocytes (Najm et al., 2013), or simple tomato lectin staining (6 μg/μl, 24 h incubation, Vector laboratories, DL-1177) for microglia (Villacampa et al., 2013). Sections were washed in 50 mM TBS-T for 1 h and incubated for 2 h in 50 mM TBS-T containing 2% BSA, the anti-rabbit Alexa Fluor 488-conjugated secondary antibody (1:400, Invitrogen, A21441) and the anti-donkey Alexa Fluor 555-conjugated secondary antibody (1:400, Invitrogen, A21432) for DYN, PV, CR and NPY staining or anti-mouse Alexa Fluor 555-conjugated secondary antibody (1:400, Invitrogen, A31570) for DRD2, NeuN, GFAP and MBP staining. After an additional wash in 50 mM TBS for 1 h, sections were mounted onto gelatine-coated slides and coverslipped using Vectashield (Vector Laboratories, H-1000).

### Image analysis

For each immunohistochemical marker, we took representative images with a laser scanning confocal microscope (Zeiss LSM 510 Meta) at a magnification of 100×. For the quantitative analysis, we used another fluorescence microscope interfaced to a computer (Zeiss Axioplan 2 Imaging). Pictures were made at a magnification of 20× in the areas of interest. Double immunohistochemistry images were processed using Fiji software (fiji.sc/Fiji). The researcher who did the counting was blind to the different treatments and the counting of the positive nuclei in the unprocessed (i.e., raw) images was conducted offline using the Fiji cell counter plugin. Immunoreactivity is given as immunopositive cells per mm^2^. Sections co-labeled for cholinergic interneurons or GABAergic interneurons, or different glia markers were not quantified, as no colocalization of EGR1 with any of these markers was observed. To investigate colocalization of the glial markers GFAP and MBP a z-stack was taken with the confocal microscope.

### Data analysis

All results are presented as the group mean ± standard error of the mean (SEM) of *n* individual animals (naïve, *n* = 8; NONCONT, *n* = 5; COCAINE, *n* = 6; and SOCIAL, *n* = 8). For each individual animal, the counts for all three slices per immunohistochemical double staining were averaged before being further processed as one value per animal. The total EGR1 signal for MSNs was obtained by pooling data from the three DYN+EGR1 slices and the three DRD2+EGR1 slices per animal. Times in the three compartments of the CPP apparatus or differences in EGR1 protein expression per mm^2^ were analyzed using a One-Way ANOVA with Tukey *post-hoc* comparison. Activation of D1- and D2-MSNs (expressed as EGR1 ir nuclei as percent of neuron type) in the COCAINE and SOCIAL group was compared using an unpaired one-tailed *t*-test. Correlations are expressed as Spearman's rank correlation coefficients (one-tailed *p*-values). All statistical tests were performed using GraphPad Prism 4 (www.graphpad.com).

## Results

### Social interaction counterconditioning inhibits reacquisition of cocaine CPP

**Figure 2:**
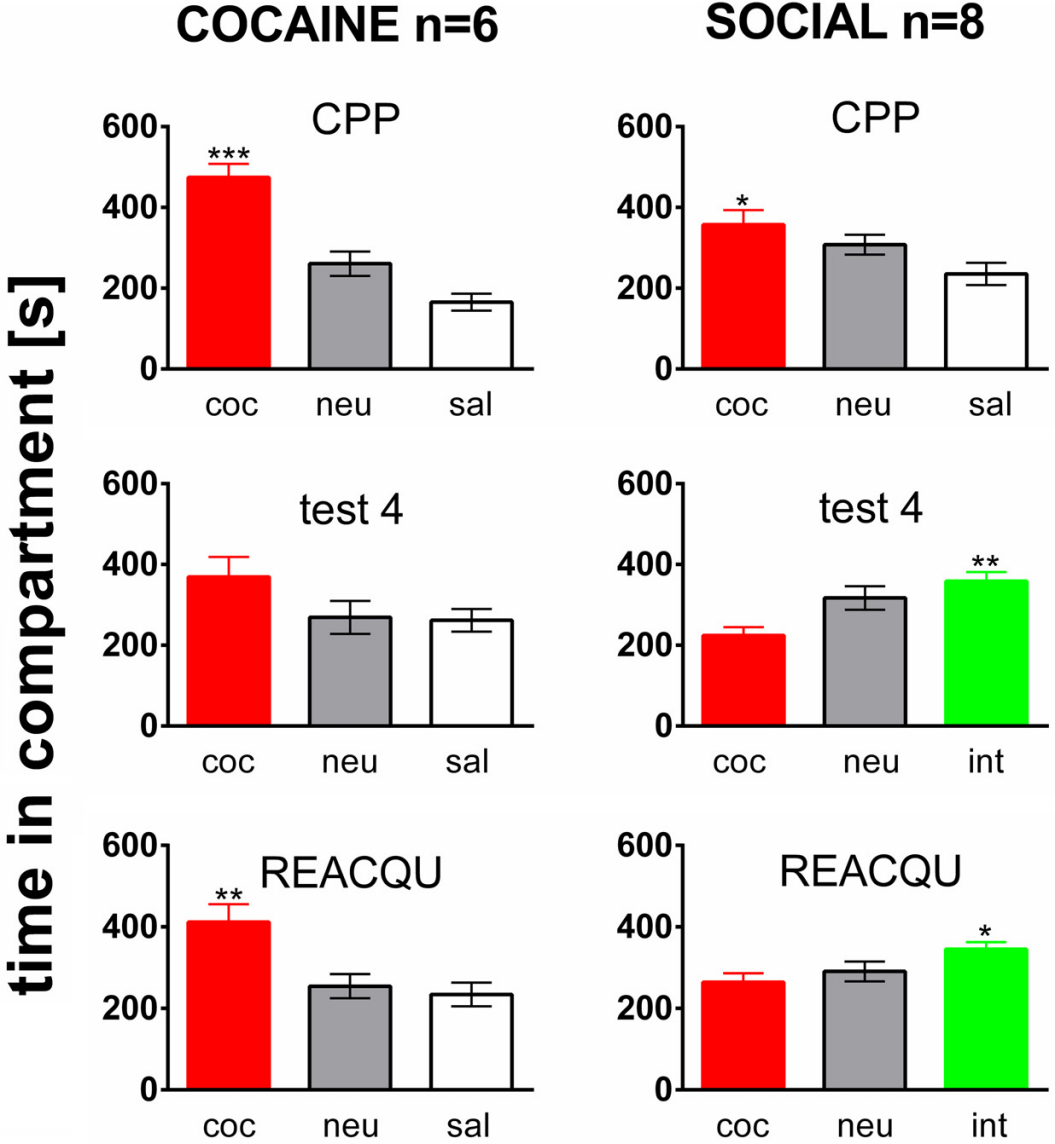
**Social interaction during extinction prevents reacquisition of cocaine CPP**. Shown are times spent in the different compartments of the CPP box, i.e., the cocaine- (coc), neutral (neu), or saline- (sal) associated compartment, or the saline compartment where social interaction counterconditioning took place (int). Time in the compartment is presented in seconds as the group mean ± s.e.m. Total session duration was 900 s. Significant differences between the time spent in the coc compartment and the time spent in the sal or int compartment are shown as an asterisk: ^*^p < 0.05; ^**^p < 0.01; ^***^p < 0.001 (One-Way ANOVA). Data are shown for the Sprague–Dawley rats of the COCAINE and SOCIAL groups for the initial cocaine CPP test (CPP, top row), for the test at the end of the fourth extinction/counterconditioning cycle (test 4, middle row), i.e., at the end of either a cocaine CPP extinction treatment with saline (COCAINE group) or a social interaction counterconditioning procedure (SOCIAL group), and during the final cocaine CPP reacquisition test (performed 24 h after a final cocaine reexposure, i.e., in a cocaine-free state).

Rats of both the COCAINE and SOCIAL group developed a cocaine CPP for the cocaine associated compartment (Figure 2, top row, CPP). This is in line with previous results of our laboratory (Fritz et al., 2011b). The preference for the cocaine associated compartment was decreased (i.e., partially extinguished) in the COCAINE group over four extinction cycles (middle row, test 4, COCAINE). If social interaction counterconditioning in the previous saline associated compartment was available during cocaine CPP extinction in the SOCIAL group, the preference shifted from the cocaine associated compartment to the social interaction associated one, i.e., social interaction counterconditioning was observed (test 4, SOCIAL). If cocaine CPP extinction was followed by only one pairing session of cocaine in the cocaine compartment, the preference for the cocaine associated compartment was reacquired (REACQU, COCAINE). In contrast, the rats shown in the SOCIAL group did not reacquire a preference for the cocaine associated compartment (REAQU, SOCIAL), instead persisting in their preference for the social interaction associated compartment as demonstrated previously (Fritz et al., 2011b).

### Cocaine CPP reacquisition-induced EGR1 expression is restricted to D1- and D2-medium spiny neurons

**Figure 3:**
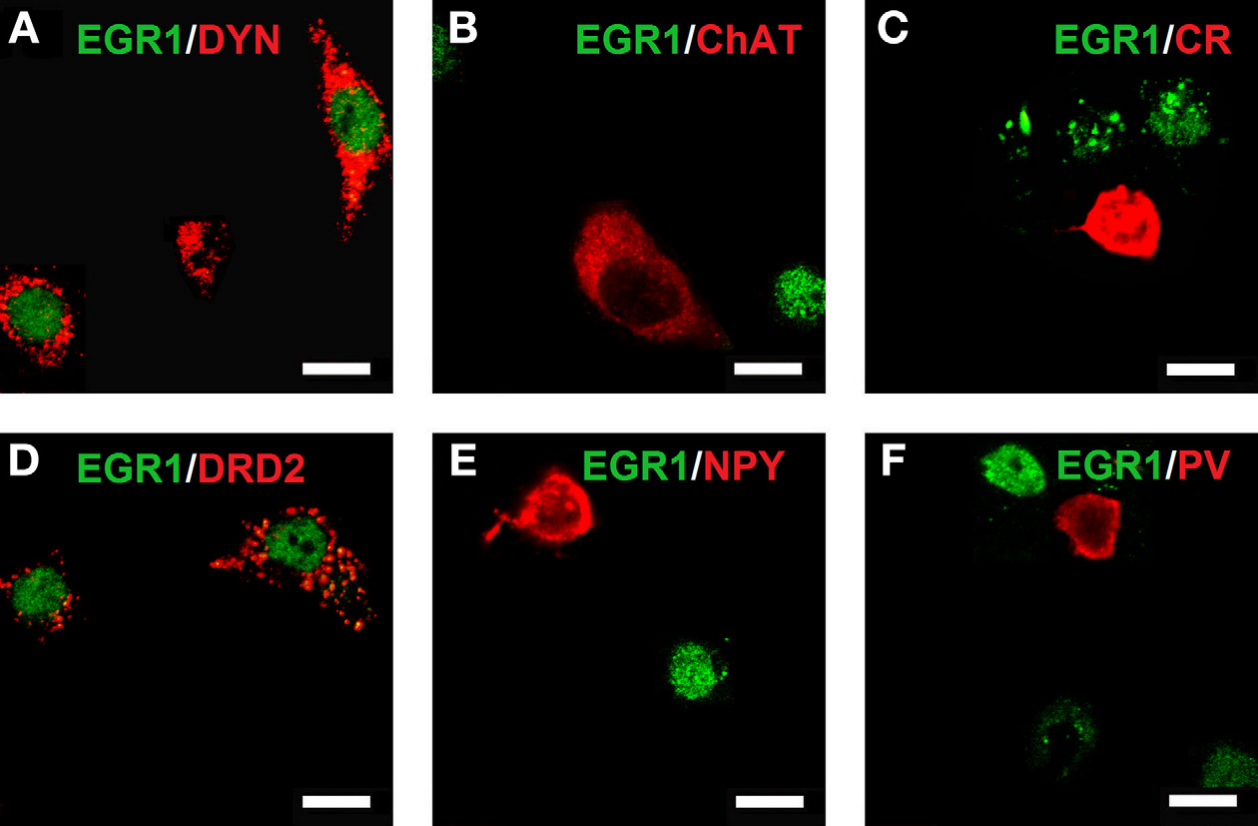
**Colocalization of neuronal markers with EGR1 expression 2 h after cocaine CPP reacquisition**. Brains were harvested and fixed for double fluorescence immunohistochemistry 2 h after the start of a 15-min cocaine CPP reacquisition test session. EGR1 immunoreactivity (green) remained restricted to nuclei. All other neuronal markers are shown in red. Colocalization of immunoreactivity was found only in medium spiny neurons (MSNs) positive for dynorphin **(A)** or the dopamine D2 receptor **(D)**. No colocalization with EGR1 was observed in cholinergic interneurons (marker: choline acetyltransferase; **(B)** or GABAergic interneurons positive for calretinin **(C)**, neuropeptide Y **(E)**, or parvalbumin **(F)**. Images were taken with a confocal laser scanning microscope with a magnification of 100× (bar size, 10 μm).

**Figure 4:**
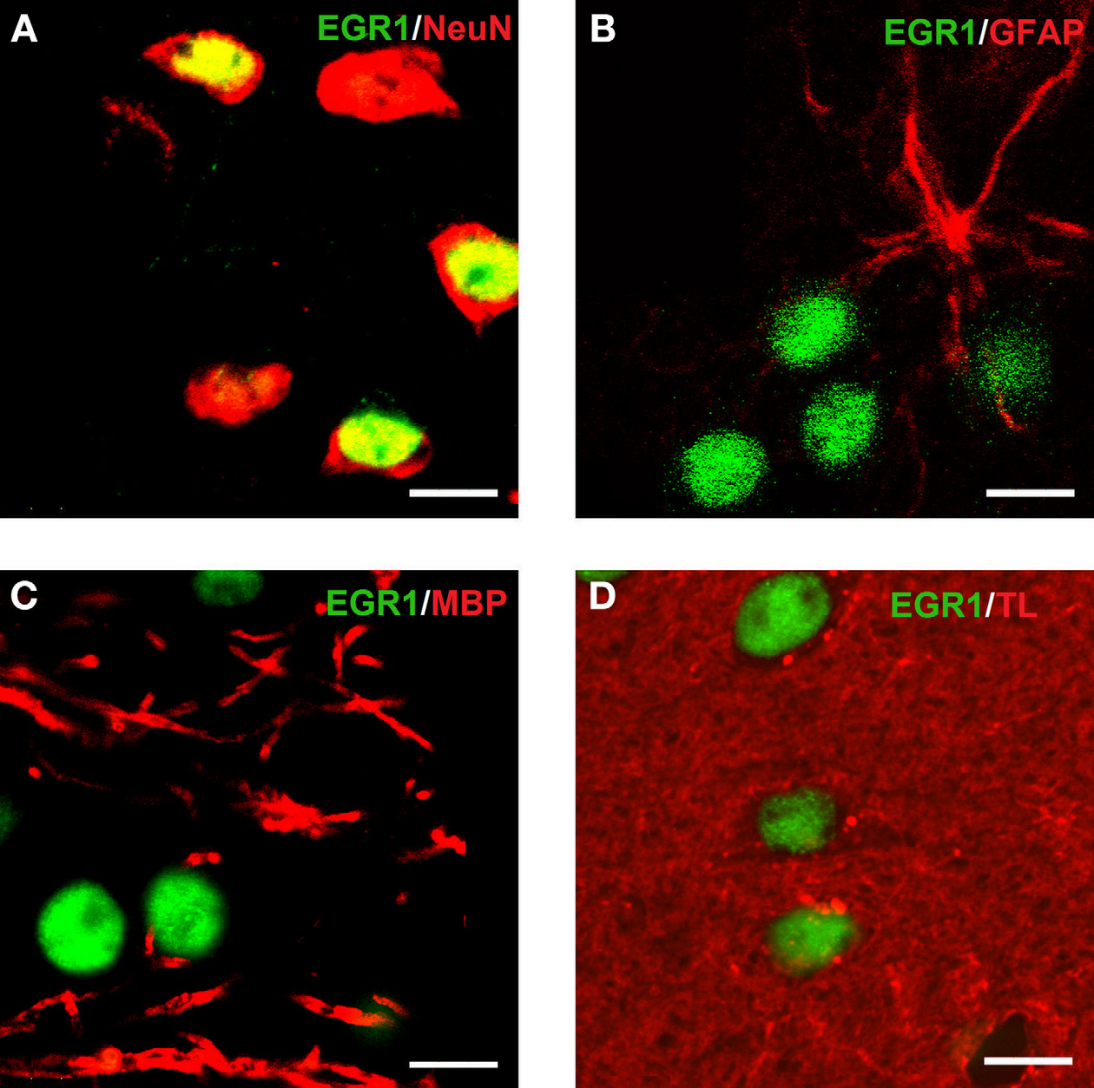
**Colocalization of EGR1 with the neuronal marker NeuN but not with glial markers**. EGR1 immunoreactivity is shown in green, all other markers are shown in red. Colocalization of immunoreactivity was found only in cells positive for the neuronal nuclear protein NeuN **(A)**. No colocalization with EGR1 was observed in glial cells. The employed glial markers were glial fibrillary acidic protein for astrocytes **(B)**, myelin basic protein for oligodendrocytes **(C)**, and tomato lectin for microglia **(D)**. Images were taken with a laser scanning confocal microscope with a magnification of 100× (bar size, 10 μm).

**Figure 5:**
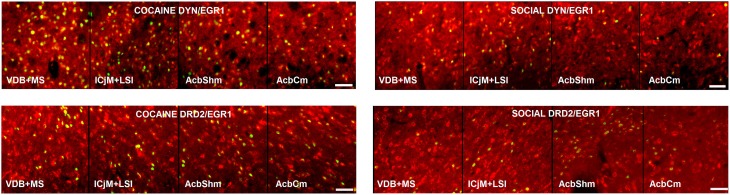
**Activation of the accumbens corridor by cocaine CPP and its inhibition by previous social interaction counterconditioning**. Shown are the merged images of EGR1- and DYN-labeled neurons, presumably D1-MSNs, or EGR1- and DRD2-labeled neurons in animals that were conditioned for cocaine and extinguished with saline (COCAINE, left panel) or animals that were conditioned for cocaine followed by social interaction counterconditioning (SOCIAL, right panel). Bar size, 50 μm.

Reacquisition of cocaine CPP induced an increase in EGR1 expression that remained restricted to cells immunopositive for dynorphin, presumably representing dopamine D1 receptor-expressing medium spiny neurons (D1-MSNs), and to cells immunopositive for the dopamine D2 receptor gene DRD2 product, i.e., D2-MSNs (Figure 3). No colocalization could be observed for EGR1 with markers for cholinergic interneurons (choline acetyltransferase) or GABAergic interneurons (CR, PV, NPY; Figure 3) in any of the treatment groups, suggesting that these neuron types are not involved in mediating cocaine CPP induced EGR1 expression or its inhibition by social interaction. We also investigated the involvement of glial cells in the observed EGR1 activation: No colocalization could be observed for EGR1 with markers for glial cells (GFAP, MBP or tomato lectin) in any of the treatment groups (Figure 4), suggesting that glial cells are not involved in mediating the observed behavioral changes. In contrast, every nucleus that was immunoreactive for EGR1 also showed colocalization with the neuronal marker NeuN (Figure 4A). Cocaine CPP reacquisition-induced EGR1 expression in the COCAINE group (Figure 5, left panel) and SOCIAL group (Figure 5, right panel) occurred in both D1-MSNs and D2-MSNs in the accumbens corridor.

### Cocaine CPP reacquisition-induced EGR1 expression in the whole accumbens corridor is inhibited by previous social interaction counterconditioning

**Figure 6:**
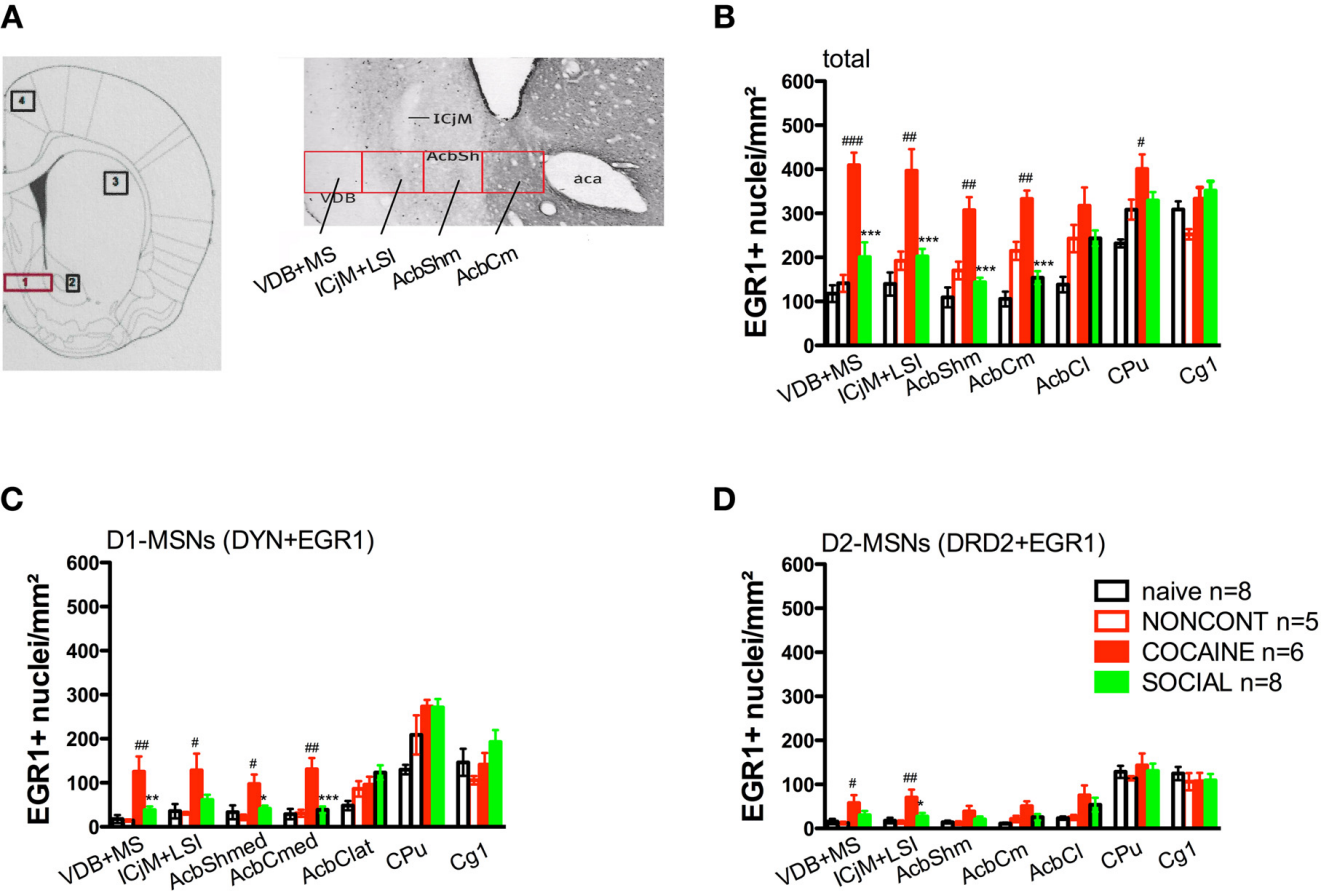
**Social interaction reverses cocaine CPP reacquisition-induced EGR1 expression in the whole accumbens corridor**. Panel **(A)** shows a diagram taken from the brain atlas of Paxinos and Watson (2007) on the left side. The numbers refer to the following regions: 1, accumbens corridor; 2, AcbCl; 3, CPu; and 4, Cg1. On the right side of **(A)**, the individual 250 μm counting bins of the different accumbens corridor regions are overlaid on a calbindin staining at an AP location of ±0.992 mm from Bregma (modified from Paxinos et al., 2009). Abbreviations (see Materials and Methods) follow the nomenclature of the atlas of Paxinos and Watson (2007). Panels **(B–D)** show group mean ± s.e.m. of EGR1-positive nuclei per mm^2^ **(B)** or the number of EGR1-positive D1-MSNs (DYN-positive, **C**) or EGR1-positive D2-MSNs (DRD2-positive, **D**) in the respective brain areas. For reasons of clarity, significant differences between treatment group are only given for the comparison of NONCONT vs. COCAINE (^#^*p* < 0.05; ^##^*p* < 0.01; ^###^*p* < 0.001) and COCAINE vs. SOCIAL (^*^p < 0.05; ^**^p < 0.01; ^***^p < 0.001). For the complete statistical analysis, see Table 1.

Two hours after the cocaine CPP reacquisition test, EGR1 expression was significantly increased in COCAINE animals compared to NONCONT animals in the whole accumbens corridor and the CPu, but not in the AcbCl or the Cg1 (Figure 6B). Statistical data for the various comparisons are shown in Table 1. Social interaction inhibited the cocaine CPP induced EGR1 activation in the whole accumbens corridor, whereas brain regions lateral of the anterior commissure (AcbCl and CPu) showed less activation in COCAINE animals and no significant inhibition by social interaction (Figure 6B). The designated negative control region, i.e., Cg1, showed no change by either COCAINE or SOCIAL (Figure 6B).

**Table 1:** **Cocaine CPP-induced EGR1 expression is reversed by social interaction in the whole accumbens corridor and more in D1-MSNs than D2-MSNs: Statistical evidence**.

	**Accumbens corridor**						
**Treatments**	**VDB+MS**	**ICjM+LSI**	**AcbShm**	**AcbCm**	**AcbCl**	**CPu**	**Cg1**
**COCAINE vs. NAÏVE**							
Total	*p* < 0.001	*p* < 0.001	*p* < 0.001	*p* < 0.001	*p* < 0.001	*p* < 0.001	n.s.
D1-MSNs	*p* < 0.001	*p* < 0.05	*p* < 0.05	*p* < 0.001	n.s.	*p* < 0.001	n.s.
D2-MSNs	*p* < 0.05	*p* < 0.01	n.s.	*p* < 0.01	n.s.	n.s.	n.s.
**COCAINE vs. NONCONT**							
Total	*p* < 0.001	*p* < 0.01	*p* < 0.01	*p* < 0.01	n.s.	*p* < 0.05	n.s.
D1-MSNs	*p* < 0.01	*p* < 0.05	*p* < 0.05	*p* < 0.01	n.s.	n.s.	n.s.
D2-MSNs	*p* < 0.05	*p* < 0.01	n.s.	n.s.	n.s.	n.s.	n.s.
**COCAINE vs. SOCIAL**							
Total	*p* < 0.001	*p* < 0.001	*p* < 0.001	*p* < 0.001	n.s.	n.s.	n.s.
D1-MSNs	*p* < 0.01	n.s.	*p* < 0.05	*p* < 0.001	n.s.	n.s.	n.s.
D2-MSNs	n.s.	*p* < 0.05	n.s.	n.s.	n.s.	n.s.	n.s.
**NONCONT vs. SOCIAL**							
Total	n.s.	n.s.	n.s.	n.s.	n.s.	n.s.	*p* < 0.05
D1-MSNs	n.s.	n.s.	n.s.	n.s.	n.s.	n.s.	n.s.
D2-MSNs	n.s.	n.s.	n.s.	n.s.	n.s.	n.s.	n.s.
**NONCONT vs. NAÏVE**							
Total	n.s.	n.s.	n.s.	*p* < 0.01	n.s.	n.s.	n.s.
D1-MSNs	n.s.	n.s.	n.s.	n.s.	n.s.	n.s.	n.s.
D2-MSNs	n.s.	n.s.	n.s.	n.s.	n.s.	n.s.	n.s.
**SOCIAL vs. NAÏVE**							
Total	n.s.	n.s.	n.s.	n.s.	*p* < 0.05	*p* < 0.01	n.s.
D1-MSNs	n.s.	n.s.	n.s.	n.s.	*p* < 0.01	*p* < 0.001	n.s.
D2-MSNs	n.s.	n.s.	n.s.	n.s.	n.s.	n.s.	n.s.

*EGR1 expression was compared by One-Way ANOVA and Tukey post-hoc test between the following treatment groups: COCAINE (n = 6), naïve (n = 8), NONCONT (n = 5), and SOCIAL (n = 8) in the following brain regions: the accumbens corridor (VDB+MS, ICjM+LSI, AcbShm, and AcbCm), the AcbCl, CPu, and Cg1. For a definition of the abbreviations see Materials and Methods*.

### The cocaine CPP reacquisition-induced EGR1 activation and its inhibition by social interaction is mediated by D1-MSNs and D2-MSNs

The number of EGR1- and DYN-positive neurons (D1-MSNs) per mm^2^ was increased in animals with a previous cocaine history (COCAINE) compared to NONCONT animals in the whole accumbens corridor: VDB+MS (*p* < 0.01), ICjM+LSI (*p* < 0.05), AcbShm (*p* < 0.05), AcbCm (*p* < 0.01; Figure 6C). Previous social interaction reversed the cocaine CPP reacquisition-induced EGR1 activation in D1-MSNs in nearly all areas of the accumbens corridor, i.e., VDB+MS (*p* < 0.01), AcbShm (*p* < 0.05), AcbCm (*p* < 0.001) but not in lateral regions, i.e., AcbCl and the CPu. Region Cg1 also did not show any significant difference in the activation of D1-MSNs. The number of EGR1 positive D2-MSNs per mm^2^ in COCAINE animals was significantly different from the NONCONT group in the VDB+MS (*p* < 0.05) and the ICjM+LSI (*p* < 0.01). Previous social interaction (SOCIAL) decreased the number of EGR1 positive D2-MSNs only in the ICjM+LSI (*p* < 0.05) as shown in Figure 6D.

### D1-MSNs are preferentially affected by the cocaine CPP reacquisition-induced EGR1 activation and its inhibition by previous social interaction in the whole accumbens corridor

**Figure 7:**
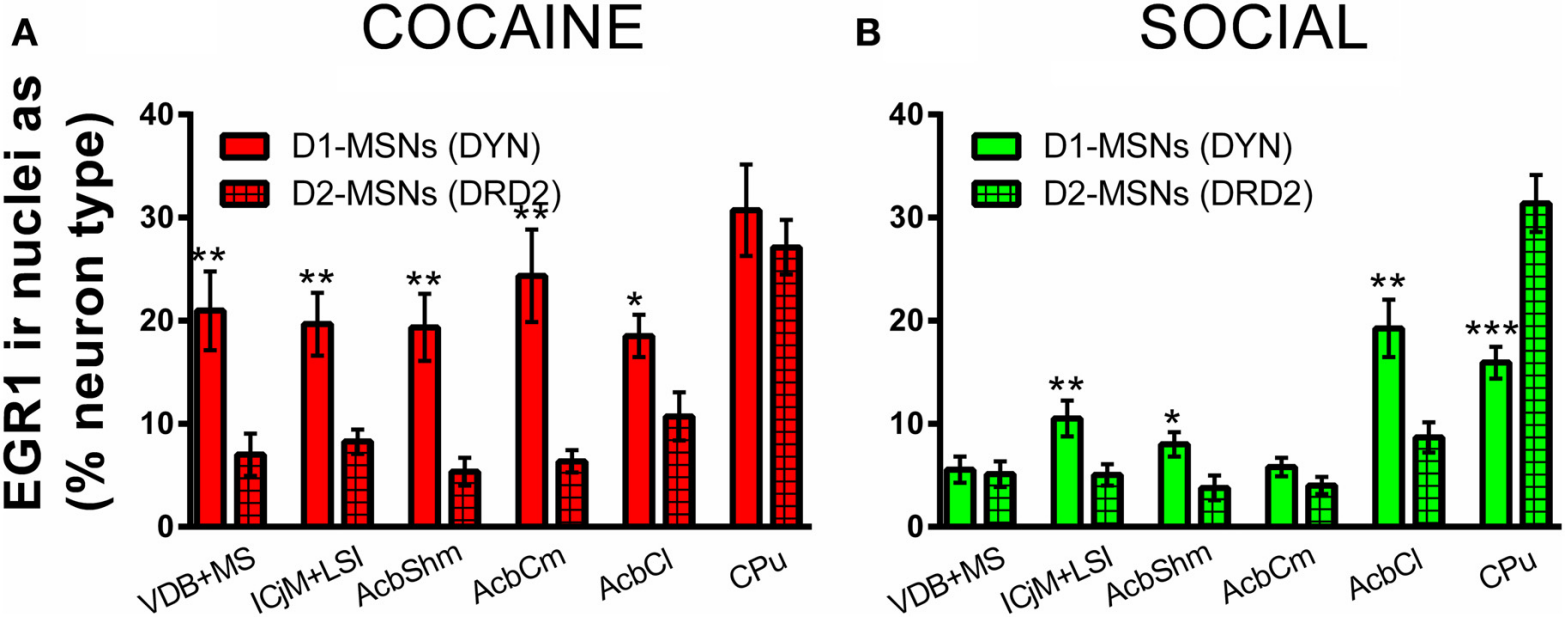
**Cocaine CPP-induced EGR1 expression is reversed by social interaction preferentially in D1-MSNs**. Shown are means ± s.e.m.'s of EGR1-immunoreactive nuclei expressed as percent of dynorphin-labeled cells, presumably D1-MSNs (open bars) vs. D2-MSNs labeled with the dopamine D2 receptor (grid-pattern bars) for the accumbens corridor regions, the AcbCl, and the CPu. Panel **(A)** (red bars) shows the percent of activated D1-MSNs and D2-MSNs for the COCAINE group, whereas panel **(B)** (green) those for the SOCIAL group. ^*^p < 0.05; ^**^p < 0.01; ^***^p < 0.001.

Throughout the accumbens corridor, dynorphin-immunoreactive cells, i.e., presumably D1 receptor expressing MSNs were activated (i.e., showed EGR1 expression) at a higher percentage than D2-MSNs (labeled with DRD2) by the cocaine associated contextual cues during the CPP reacquisition test (Figure 7A). In animals previously exposed to social interaction, cocaine CPP reacquisition induced EGR1 expression was generally lower (Figure 7B) than in saline-extinguished animals (Figure 7A) across both neuron types (D1- and D2-MSNs).

### EGR1 expression correlates with the time in the cocaine compartment in the whole accumbens corridor

**Figure 8:**
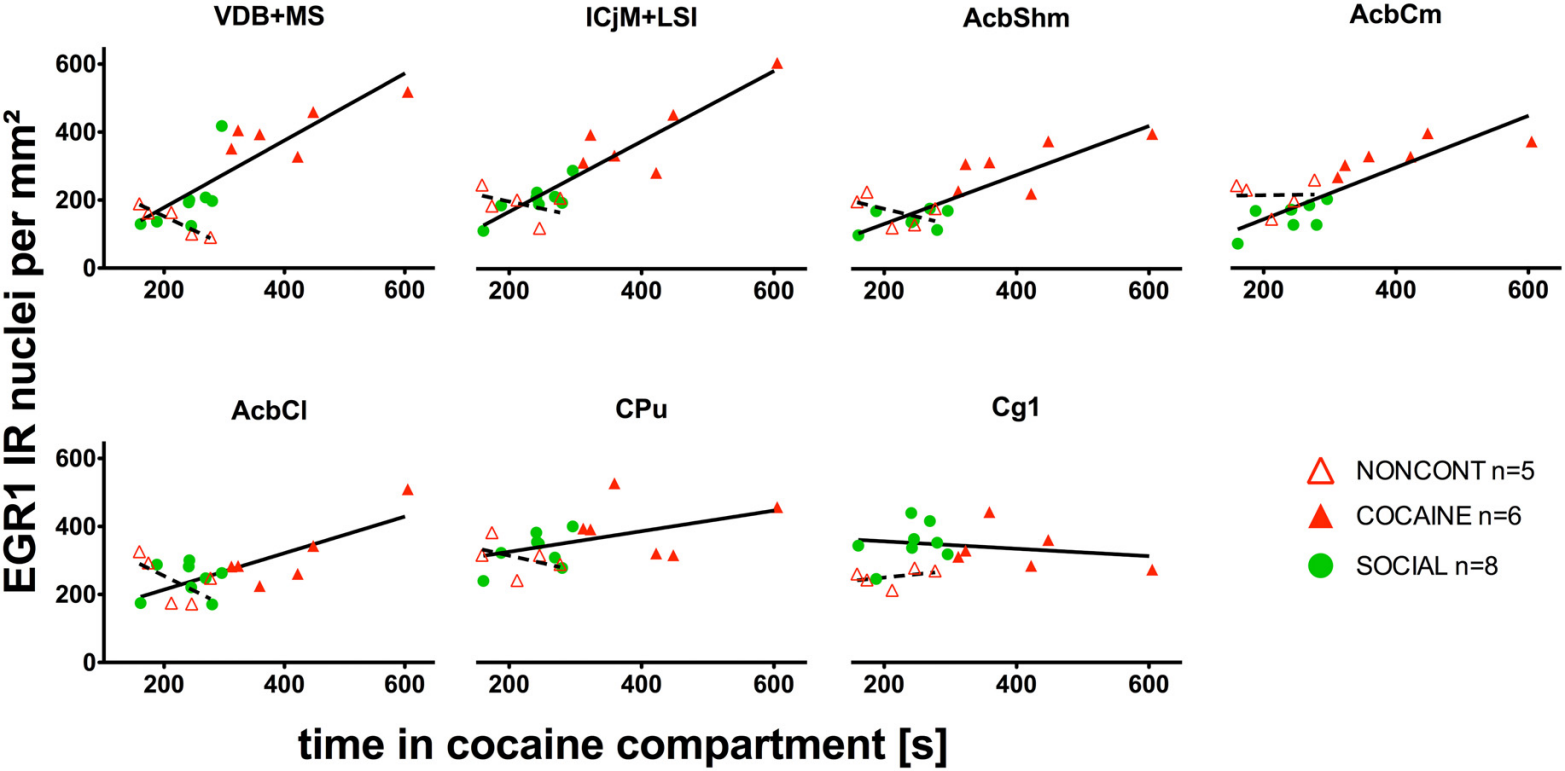
**EGR1 expression in the whole accumbens corridor 2 h after cocaine CPP reacquisition correlates with time spent in the cocaine compartment**. The correlation of EGR1 expression per mm^2^ vs. the time spent in the cocaine compartment during the cocaine CPP reacquisition test is given for animals that had undergone cocaine conditioning and extinction with saline (COCAINE, red triangles, filled) and for animals that were counterconditioned with social interaction after an initial cocaine conditioning (SOCIAL, green circles, filled) and is shown as a continuous line. There was no statistically significant correlation (shown as a dashed line) between the EGR1 expression per mm^2^ and the time spent in the cocaine compartment for animals injected noncontingently with cocaine (NONCONT; red triangles, unfilled).

We investigated if there was a correlation between the animals' preference for the cocaine-associated contextual cues (as quantified in the CPP paradigm) and neuronal activation in the accumbens corridor regions (as determined by EGR1 activation). As we wanted to test if this is a general phenomenon (i.e., independent of variations in conditioning), we pooled the data from the two conditioning groups, following field precedence (Golden et al., 2013). The respective correlational statistics for the individual treatment groups are given at the end of this paragraph. For the pooled conditioning groups, the number of EGR1 positive nuclei per mm^2^ was indeed significantly correlated (Figure 8) with the time spent in the cocaine compartment for data pooled from both the COCAINE and SOCIAL group: VDB+MS (*r* = 0.86, *p* < 0.0001), ICjM+LSI (*r* = 0.89, *p* < 0.0001), AcbShm (*r* = 0.87, *p* < 0.0001), AcbCm (*r* = 0.9, *p* < 0.0001), but not in the AcbCl (*r* = 0.33, *p* = 0.12), the CPu (*r* = 0.41, *p* = 0.07), or the Cg1 (*r* = −0.16, *p* = 0.29). There was no statistically significant correlation between EGR1 expression and time spent in the cocaine compartment for animals injected NONCONT in the ICjM+LSI (*r* = −0.3, *p* = 0.34), AcbShm (*r* = −0.5, *p* = 0.23), AcbCm (*r* = 0.1, *p* = 0.48), AcbCl (*r* = −0.7, *p* = 0.12), CPu (*r* = −0.6, *p* = 0.18), and Cg1 (*r* = 0.5, *p* = 0.23); but see the VDB+MS (*r* = −0.9, *p* = 0.04). The Spearman's rank correlation coefficients and *p*-values for the individual treatment groups were, for the COCAINE group and the various brain regions: VDB+MS, *r* = 0.6, *p* = 0.12; ICjM+LSI, *r* = 0.6, *p* = 0.12; AcbShm, *r* = 0.66, *p* = 0.088; AcbCm, *r* = 0.89, *p* = 0.017; AcbCl, *r* = 0.54, *p* = 0.15; for CPu, *r* = −0.14, *p* = 0.4; and for Cg1, *r* = −0.3, *p* = 0.28. SOCIAL group: VDB+MS, *r* = 0.67, *p* = 0.04; ICjM+LSI, *r* = 0.69, *p* = 0.035; AcbShm, *r* = 0.45, *p* = 0.13; AcbCm, *r* = 0.52, *p* = 0.099; AcbCl, *r* = −0.24, *p* = 0.29; CPu, *r* = 0.31, *p* = 0.23; and for Cg1, *r* = −0.07, *p* = 0.44. NONCONT COC group: VDB+MS, *r* = −0.9, *p* = 0.042; ICjM+LSI, *r* = −0.3, *p* = 0.34; for AcbShm, *r* = −0.5, *p* = 0.23; for AcbCm, *r* = 0.1, *p* = 0.48; AcbCl, *r* = −0.7, *p* = 0.12; CPu, *r* = −0.6, *p* = 0.18; and Cg1, *r* = 0.5, *p* = 0.23.

## Discussion

The present study yielded two major findings: (1) The degree of reacquisition of cocaine CPP in our animal model of cocaine relapse is proportional to the activation of medium spiny neurons, predominantly D1-MSNs, in all regions of a corridor extending mediolaterally from the interhemispheric border to the anterior commissure. (2) This neuronal activation is greatly inhibited by a previous history of counterconditioning with social interaction.

Our experimental paradigm (Zernig et al., 2013) quantifies the renewed intensification of the incentive salience of cocaine-associated contextual stimuli (Zernig et al., 2007). Because an increase in the incentive salience of drug-associated stimuli very likely leads to a relapse in humans (Epstein et al., 2009), our animal model has translational power with respect to cocaine relapse in humans and opens the way for the neurobiological investigation of the beneficial effects of dyadic (i.e., one-to-one) social interaction between sex- and weight-matched conspecifics on subsequent cocaine craving and consumption.

With respect to age, the male Sprague–Dawley rats in our paradigm (6–8 weeks upon arrival) can be considered “early adult” (Spear, 2000; Zernig et al., 2013). We had originally chosen this age based on the simplistic notion of one of us (Gerald Zernig) that at a younger age, individuals of any species will find the interaction with a conspecific more rewarding than older individuals do, thus increasing the likelihood of developing CPP for dyadic social interaction in our paradigm (Zernig et al., 2013). Addressing this notion, Bardo and coworkers (Yates et al., 2013) systematically investigated the effect of age in our concurrent CPP paradigm, pitching d-amphetamine (1 mg/kg s.c.) vs. dyadic social interaction and found that “young” male Sprague–Dawley rats (3 weeks upon arrival) developed a preference for social interaction, whereas “adult” rats (8.5 weeks upon arrival) preferred the drug over social interaction in this paradigm. To summarize these findings, dyadic social interaction seems more rewarding for adolescent and early adult rats than for older ones. Accordingly, gross visual inspection of our animals showed that the early adult rats frequently engaged in play behavior (Trezza et al., 2010) and other “friendly” (“agonistic”) interaction (data not shown).

It may be argued that social interaction itself is not the factor that alters gene expression/preference or just simply the fact that something besides the rat itself is present in the non-cocaine-associated compartment. However, when carefully separating the composite stimulus “social interaction” into its sensory components, we found that rat odor emitting bedding (olfaction) alone or sensing another rat through a see-through screen via the vibration of the other moving rat, its smell, its sight and its sound proved much less rewarding than having direct tactile contact and the possibility to interact with the other rat through prison-type bars (Kummer et al., 2011). Accordingly, if the social interaction space was decreased by half, i.e., if crowding was induced experimentally, the social interaction reward was considerably reduced (Kummer et al., 2011). All these findings suggest that is it the social interaction—and not the sensory stimulation associated with it—that causes CPP in our paradigm.

Our choice of EGR1 as a brain activation marker was based on the seminal paper by Everitt and coworkers (Lee et al., 2005) who showed that EGR1 antisense oligodeoxynucleotides abolished the acquired conditioned reinforcing properties of a cocaine-associated stimulus (Zernig et al., 2013).

With respect to the immunohistochemical marker used to label D1-MSNs, i.e., dynorphin, it must be noted that dynorphin is a reliable marker for D1-MSNs in the dorsal striatum and the ventral striatum (Curran and Watson, 1995; Fricks-Gleason and Marshall, 2011). Additionally it is clearly shown that D1 and D2 class receptors in the dorsal and ventral striatum are principally expressed in separate neuron populations (Le Moine et al., 1990; Le Moine and Bloch, 1991, 1995, 1996; Curran and Watson, 1995; Steiner and Gerfen, 1998). However, in the regions more medial of the AcbSh not much data have been generated to prove the coexpression of dopamine D1 receptor and dynorphin. Thus, the question of reliability of using dynorphin as a marker for D1 receptor expressing neurons in these specific medial regions has to be addressed in our future studies.

The homogeneity of the cocaine CPP reacquisition-induced EGR1 response of such diverse structures as the nucleus of the vertical limb of the diagonal band, the medial septal nucleus, the major island of Calleja, the intermediate part of the lateral septal nucleus, and the medial accumbal shell and core is striking. Nonspecifity of the observed differential activation pattern during reacquisition of cocaine CPP in saline-extinguished vs. social interaction-counterconditioned animals and nonspecifity of the proportionality between cocaine CPP preference and EGR1 activation can be excluded as the negative control region, i.e., the anterior cingulate area Cg1, did not display a similar differential activation pattern or proportionality between cocaine preference and EGR1 activation. Of interest, the AcbCl, i.e., the core region lateral of the anterior commissure, which is contiguous with the CPu proper, and the dorsal CPu also failed to show a statistically significant correlation between cocaine CPP preference and EGR1 activation while yielding a similar but less pronounced pattern compared to the regions of the accumbens corridor, strongly supporting the concept of a (ventro)medial to (dorso)lateral striatal gradient for the expression of motivated behavior toward various stimuli (Ikemoto, 2007; Haber and Knutson, 2010).

Why would such apparently different (Zahm and Heimer, 1993) brain regions show such a homogeneous response? The most parsimonious explanation is that they share the same inputs. Indeed, the medium spiny neurons which are the predominant neuron type in all the regions of the accumbens corridor (Ribak and Fallon, 1982; Fallon et al., 1983; Guo et al., 1998; Rotllant and Armario, 2012; Zahm et al., 2013)—be they called “granule cells” in the ICjM or “MSNs” in the Acb (Ribak and Fallon, 1982)—almost all receive direct projections from the hippocampus (Meibach and Siegel, 1977; Swanson and Cowan, 1979; Nyakas et al., 1987; Oades and Halliday, 1987; Brog et al., 1993; Kalivas et al., 1993) and the ventral tegmental area (Fallon et al., 1978; Oades and Halliday, 1987; Kalivas et al., 1993; Fitch, 2006; Ikemoto, 2007; Mahler and Aston-Jones, 2012). The hippocampus could thus provide a major glutamatergic input to most MSNs in the accumbens corridor. The VTA also sends direct projections, most likely dopaminergic ones, to all regions in the corridor, resulting in a modulation of the D1- vs. D2-receptor-carrying MSNs.

Both D1- and D2-MSNs are known to form dendritic and axonal spheres with diameters of approximately 200 μm (Gerfen, 2004; Humphries et al., 2010), interconnecting and displaying collateral inhibition (Witten et al., 2010) across the regional borders of the accumbens corridor (Zahm et al., 2013): The LSI, for example, forms strong reciprocal connections with the VDB, MS, and AcbShm (Zahm et al., 2013). The ICjM, as part of the island of Calleja complexes (Ribak and Fallon, 1982), receives projections from, among others, the ventral pallidum (VP), septum, cortical nuclei of the amygdala, lateral hypothalamic area, ventral tegmental area, the hypothalamus, and midline, intralaminar and medial thalamic nuclei. These connections are consistent with the concept that the ICjM, as part of the ICC, is a striato-pallidal structure (Fallon et al., 1983). The similarities of the input to the ICjM and a seemingly very different region in the accumbens corridor, i.e., the Acb proper, are striking. Our findings strengthen the view (Zahm, 2008; Zahm et al., 2013) that “accumbal” histoarchitectonics and functionality are not limited to an Acb “nucleus” but that a continuum exists between what can be defined, by selected histochemical markers (Paxinos et al., 2009), as the medial “core” and “shell” of an accumbal “nucleus” and neighboring structures (Zahm, 2008). Accordingly, the accumbal subterritories at the rostral part of the AcbC and AcbSh show a lack of boundaries to neighboring regions which all have similar characteristics of morphology and immunohistochemistry (Brog et al., 1993). As a final further example, there is a strong reciprocal interconnection between the rostrodorsal part of the AcbSh and the LSI (Zahm et al., 2013).

Our results also contribute to the current intense research effort to differentiate the contribution of D1- vs. D2-MSNs to the expression of motivated behavior toward drugs of abuse vs. physiological reinforcers (Bertran-Gonzalez et al., 2008; Kravitz et al., 2012; Bock et al., 2013; Lobo et al., 2013; Maguire et al., 2014). In the dorsal striatum, D1-MSNs are part of the so-called “direct” pathway, whereas D2-MSNs belong to the “indirect” pathway (Gerfen, 2004; Wall et al., 2013). In contrast, direct and indirect pathways overlap in the nucleus accumbens as part of the ventral striatum. While D1-MSNs project to both the VP and midbrain regions (substantia nigra compacta and reticulata and ventral tegmental area), D2-MSNs only project to the VP, with the accumbens core projecting to the dorsal part of the VP and the shell projecting to the ventromedial part of the VP (Robertson and Jian, 1995). Our findings indicate that both D1- and D2-MSNs are involved in mediating the incentive salience of cocaine-associated contextual cues and its inhibition by previous social interaction but that, overall, D1-MSNs seem to be engaged to a larger degree than D2-MSNs in most areas of the accumbens corridor, corroborating findings obtained in transgenic mice (Bertran-Gonzalez et al., 2008).

Interestingly, glial cells have also been implicated in mediating various aspects of drug reward (Turner et al., 2012; Zhang et al., 2012; Schwarz et al., 2013). Accordingly, we found that the numbers of D1- and D2-MSNs immunopositive for EGR1 did not add up to the total number of EGR1-positive nuclei, suggesting (1) that we may not have immunolabeled all D1- or D2-MSNs or (2) that glial cells may have contributed to the differential changes observed in the present study. However, none of the tested glial markers yielded any double staining with EGR1. The cellular activation observed in the present behavioral experiments remained restricted to NeuN-positive cells, i.e., neurons.

How could a history of dyadic social interaction inhibit the subsequent activation of MSNs in the accumbens corridor by cocaine associated contextual stimuli? Extrapolating from the human situation as seen from the psychotherapeutic perspective, the reactivation of memories of the rewarding aspects of four social encounters with same individual (which could be reported as “positive,” soothing, and pleasurable by a human) by the contextual stimuli of the social interaction associated compartment of the three-compartment CPP could plausibly dampen the arousing and motivational effects (“craving”) induced by the contextual stimuli of the neighboring cocaine associated chamber of the CPP box as the individual moves back and forth from one conditioning chamber to the other during the (cocaine-free) CPP test. As the amygdala is known (1) to mediate memories with emotional content (Herry et al., 2010) and (2) to project extensively to the AcbShm and the AcbSh (Heimer et al., 1997), it is well conceivable that the amygdala exerts the inhibitory effect of social interaction memory on MSNs indirectly via inhibitory GABAergic interneurons (Tepper et al., 2010). The same rationale applies to the hippocampus, another well-known memory-related brain region (Meibach and Siegel, 1977; Swanson and Cowan, 1979; Nyakas et al., 1987; Oades and Halliday, 1987; Brog et al., 1993; Kalivas et al., 1993).

To summarize, the present findings support the important role of D1-MSNs in mediating the rewarding properties of drugs of abuse vs. physiological reinforcers, whereas D2-MSNs are less involved. None of the other neuron types in the accumbens corridor, i.e., cholinergic interneurons or the various GABAergic interneurons, were involved in the differential activation. In the case of the cholinergic interneurons, this was especially surprising to us, as we (Crespo et al., 2006, 2008, 2012), like many other groups (Wilson and Schuster, 1973; Acquas et al., 1996; Mark et al., 1999; Pratt and Kelley, 2004; Smith et al., 2004; Grasing et al., 2009; Witten et al., 2010; English et al., 2011; Cachope et al., 2012; De La Garza et al., 2012; Hikida et al., 2012; Threlfell et al., 2012) had provided evidence for the involvement of the accumbal cholinergic system in drug- and food reward and as cholinergic interneurons (Berlanga et al., 2003) were shown to be instrumental for the acquisition of cocaine CPP (Witten et al., 2010). Accordingly, we had demonstrated in a rat runway procedure that acetylcholine (ACh) release and activation of muscarinic and nicotinic ACh receptors were necessary for the acquisition of the rewarding properties of cocaine, of two pharmacokinetically very different μ opioid receptor agonists, i.e., remifentanil and morphine, and of highly palatable food (Crespo et al., 2006, 2008). There are at least two explanations for this apparent discrepancy: (1) the role of cholinergic interneurons diminishes after the conditioned response has been acquired, and/or (2) accumbal ACh from extra-accumbal sources (Dautan et al., 2014) becomes more important during the maintenance and reacquisition of reward.

In conclusion, our results show that D1-MSNs and to lesser degree D2-MSNs in the whole accumbens corridor medial of the anterior commissure are differentially mediated by cocaine reward vs. dyadic social interaction reward. One avenue of future research is to differentiate the neuronal ensembles (Cruz et al., 2013) in the MSN populations that modulate the attractiveness of such strikingly different types of reward.

### Conflict of interest statement

The authors declare that the research was conducted in the absence of any commercial or financial relationships that could be construed as a potential conflict of interest.
